# EnhancerPred: a predictor for discovering enhancers based on the combination and selection of multiple features

**DOI:** 10.1038/srep38741

**Published:** 2016-12-12

**Authors:** Cangzhi Jia, Wenying He

**Affiliations:** 1 Department of Mathematics, Dalian Maritime University, No. 1 Linghai Road, Dalian 116026, China

## Abstract

Enhancers are *cis* elements that play an important role in regulating gene expression by enhancing it. Recent study of modifications revealed that enhancers are a large group of functional elements with many different subgroups, which have different biological activities and regulatory effects on target genes. As powerful auxiliary tools, several computational methods have been proposed to distinguish enhancers from other regulatory elements, but only one method has been considered to clustering them into subgroups. In this study, we developed a predictor (called EnhancerPred) to distinguish between enhancers and nonenhancers and to determine enhancers’ strength. A two-step wrapper-based feature selection method was applied in high dimension feature vector from bi-profile Bayes and pseudo-nucleotide composition. Finally, the combination of 104 features from bi-profile Bayes, 1 feature from nucleotide composition and 9 features from pseudo-nucleotide composition yielded the best performance for identifying enhancers and nonenhancers, with overall Acc of 77.39%. The combination of 89 features from bi-profile Bayes and 10 features from pseudo-nucleotide composition yielded the best performance for identifying strong and weak enhancers, with overall Acc of 68.19%. The process and steps of feature optimization illustrated that it is necessary to construct a particular model for identifying strong enhancers and weak enhancers.

Transcription is mainly regulated by the binding of transcription factors (TFs) at specific DNA sequences to recruit RNA polymerase II initiation or elongation factors^1^,^2^. The most studied sites are promoter regions, which harbour transcription initiation sites. There are also some DNA sequences near or far away from promoter regions, which contain multiple transcription factor binding sites. These DNA sequences are referred to as “enhancers”^3^. The first characterized enhancer was a DNA segment that markedly increased the transcription of the β-globin gene in a transgenic assay in the SV40 tumour virus genome, about 30 years ago^4^,^5^,^6^,^7^. By enhancing the transcription of genes, enhancers influence gene expression and regulation, cell growth and differentiation, tissue specificity of gene expression, virus activity and cell carcinogenesis, and ensure the close relationship among these processes. Recent systematic genome-wide study of histone modifications has revealed that enhancers are a large group of functional elements with many different subgroups, such as strong enhancers and weak enhancers, poised enhancers and latent enhancers^3^. Understanding enhancers and their subgroups is currently an area of great interest as there is an increasing appreciation of their importance not only in developmental gene expression but also in evolution and disease^8^,^9^.

As powerful auxiliary tools, several computational prediction methods have been considered in recent years to differentiate enhancers from other regulatory elements in the genome. Various predictors have been established, such as CSI-ANN^10^, ChromiaGenSvm^11^, RFECS^12^, DELTA^13^, EnhancerFinder^14^, GKM-SVM^15^, DEEP-ENCODE^16^ and iEnhancer-2L^17^, which consider information on sequences or specific histone epigenetic marks to feature processing and integrated different classification algorithm (such as artificial neural network, support vector machine, random forest, and so on) in identifying enhancers. Note that, among all of the prediction methods, only iEnhancer-2L not only discriminates enhancers from other regulatory elements but also considers their subgroup, namely, whether they are strong or weak enhancers. iEnhancer-2L achieved overall accuracy of 76.89% for identifying enhancers and nonenhancers (denoted as layer I), and achieved overall accuracy of 61.93% for identifying strong enhancers and weak enhancers (denoted as layer II). The prediction performance of layer II was not satisfactory, so there is still room for improvement. In the present study, we first considered three types of sequence-based features (a total of 472 features) and then used the F-score to screen the optimal combination of features. Finally, 114 and 99 selected features combined with SVM were used to identify enhancers and their strength, respectively. The jackknife test results indicate that our predictor can be used as a robust tool for identifying enhancers/nonenhancers and strong enhancers/weak enhancers. For the convenience of most experimental scientists, a web-server for the predictor EnhancerPRED was available at http://server.malab.cn/EnhancerPRED/.

## Results and Discussion

### BPB feature optimization

**Figure 1:**
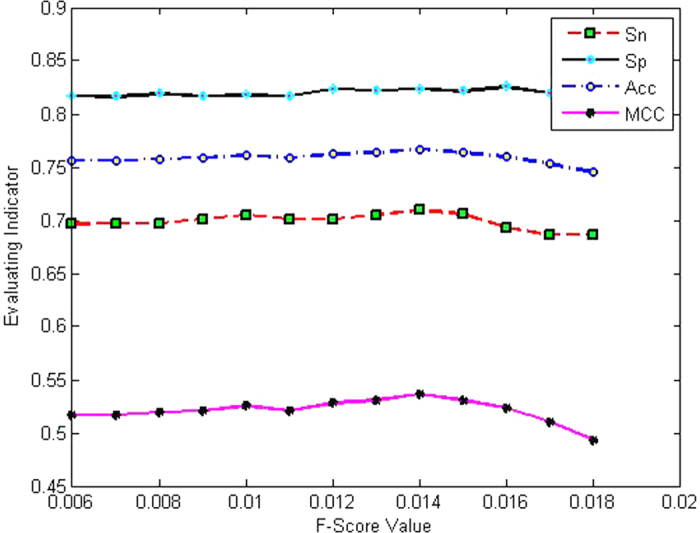
The prediction performance at different thresholds of F-score for layer I.

**Figure 2:**
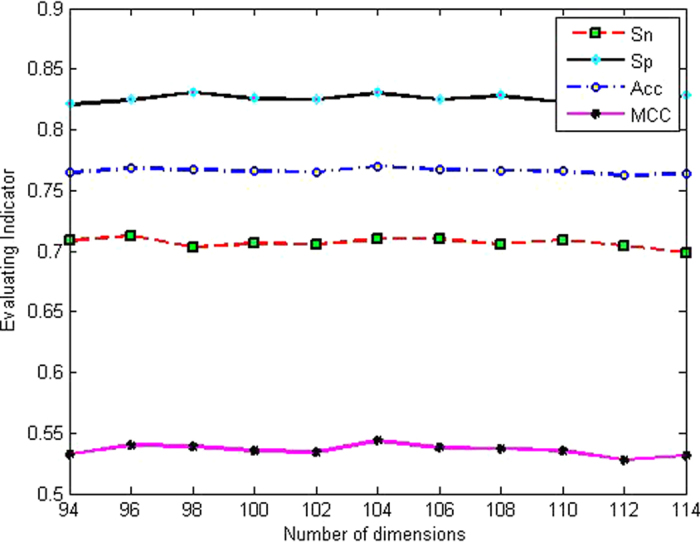
The prediction performance on different dimensions of BPB feature vector for layer I.

To remove irrelevant and redundant features and then determine the optimal combination of features, a selection method was performed using the jackknife test on the dataset. Taking the case of differentiating enhancers and nonenhancers, F-score values were first calculated to rank the 400 features derived from BPB, and then we selected those features with an F-score greater than or equal to the given threshold to establish a new predictor. The prediction performances on different F-score thresholds with intervals of Δ*w*_1_ = 0.001 are listed in Fig. 1. Acc was selected as the assessment to measure the predictor. As can be seen in Fig. 1, when the threshold of the F-score was within the range of 0.013–0.015, better Acc in the range of 76.35–76.65% was obtained. Next, we further optimized the number of dimensions of the BPB feature vector from 94 to 114 to obtain more satisfactory prediction performance. The prediction performances for different dimensions (114,112,110, …, 94) of the BPB feature vector with the step of Δ*ω*_2_ = 2 are shown in Fig. 2. As indicated in this figure, the performance achieved the best Acc of 76.99% when 104 features were selected. Therefore, an optimal number of features of 104 was retained for combination with other features to construct the optimal model.

### Combination feature optimization

F-score was also used to rank the features of NC (Table S1). First, we added the top-ranked feature from NC to the selected 104 features from BPB and then ran SVM in the jackknife cross-validation strategy. If the addition of the top-ranked feature improved the Acc, then this feature was retained; otherwise, it was removed. As shown in Tables 1 and S2, the combination of 104 BPB features and the1 NC feature reached the highest Acc of 77.02%.

As there were 64 components in PseNC, which is much more than the 4 components in NC, the process of feature selection was similar to that described in BPB feature optimization section. We used F-score to rank the 64 components of PseNC, and then selected different numbers of features according to different F-score thresholds with a step size of Δ*w*_3_ = 0.01. As illustrated in Table S3, the prediction performance first increased and then decreased, and better prediction performance was obtained in the threshold range of 0.14–0.17. Then, we performed fine screening of the number of features in PseNC from 22 to 4 with a step size of Δ*w*_4_ = 2; the detailed prediction results are shown in Table S4. Finally, by incorporating the top 9 components of PseNC with the 104 features from BPB and the 1 feature from NC, we obtained the best prediction performance with Acc of 77.39%. The increasing sequence encoding schemes are listed in Table 1.

**Figure 3:**
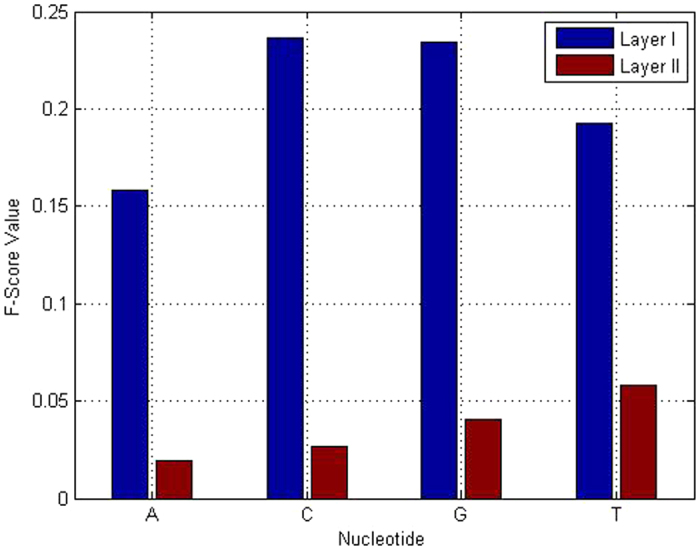
F-score values of NC in both layer I and layer II.

The same feature selection process was carried out to detect strong and weak enhancers. The detailed results are displayed in Tables S1, S5 and S6. It should be pointed out that the composition of nucleotide C contributes to the detection of enhancers and nonenhancers, but does not obviously contribute to the detection of strong enhancers and weak enhancers. As can also be seen in Fig. 3, the highest F-score reached 0.236 for enhancer and non-enhancer at the composition of nucleotide C, which means that nucleotide C was enriched in the enhancers, whereas it was depleted in the nonenhancers. However, the composition of nucleotide C exhibited no real distinction between strong and weak enhancers, having an F-score of only 0.026 (Fig. 3). We also determined that the compositions of eight 3-tuple nucleotides (‘ATA’, ‘TAT’, ‘ATT’, ‘TAA’, ‘TTA’, ‘GGC’, ‘AAT’, ‘AGG’, ‘TTT’ and ‘CAG’) are important for the identification of both layer I and layer II. This investigation also implied that the different compositions of amino acids for layers I and II justify the establishment of two predictors for detecting enhancers and nonenhancers, strong enhancers and weak enhancers, respectively.

### Comparison with other classifiers

In many fields of computational biology, k Nearest Neighbour (KNN)^18^, Naïve Bayes^19^, Random Forest (RF)^20^, Ensembles for Boosting^21^, LibD3C^22^, Gradient Boosting Decision Tree (GBDT)^23^ and SVM are the most powerful and widely used classification methods. To determine the predictors that are most effective for identifying enhancers and their strength, we compared the performances of the seven above-mentioned classifiers based on the same encoding schemes. The number of nearest neighbours will influence the performance of the KNN algorithm, and the number of trees will influence the performance of the RF algorithm. Therefore, a search was undertaken to identify the optimal parameters for RF and KNN, as shown in Tables S7 and S8, respectively.

The accuracy results in the jackknife test for the seven classifiers used are shown in Table 2. This table shows that SVM outperformed all of the other classifiers, having the highest MCC value of 0.55 for the layer I and the highest MCC value of 0.36 for layer II.

### Comparison with other methods

We used the jackknife test to evaluate our prediction model because it is considered to be the most objective as it always yields a unique result for a given dataset^24^. In this test all but one sequence in the training dataset are used to train the proposed predictor and the remaining only one sequence is used to perform the test. The jackknife test results achieved by EnhancerPred on the benchmark dataset are given in Table 3, in which the results reported by Liu *et al*.^17^ are also listed for comparison. As can be seen in this table, EnhancerPred produced greater accuracy than iEnhancer-2L, with MCC of 0.01 for the first layer and 0.12 for the second layer. This comparison indicates that the proposed predictor EnhancerPred is indeed promising or can at least play a role that complements the existing state-of-the art methods in this field^10^,^11^,^12^,^13^,^14^,^15^,^16^,^17^.

## Conclusion

Predicting the location of enhancers and the extent to which they increase gene expression is critical for obtaining a better understanding of the spatiotemporal regulation of eukaryotic gene expression. The recent accumulation of high-throughput data on enhancers has increased the demand for efficient computational approaches that are capable of accurately predicting the location of enhancers at the genome-wide level. Here, we have presented EnhancerPred, a novel bioinformatics tool that formulates the prediction of enhancers and their strength as a binary classification problem and solves it using a machine learning algorithm. This tool extracts features using BPB, NC and PseNc and also takes advantage of efficient feature selection, which was shown here to be robust and high performing using a rigorous jackknife test. In comparison to existing tools, such as iEnhancer-2L, EnhancerPred achieved satisfactory MCC values, especially for the prediction of whether an enhancer has a strong or weak effect on gene expression. For the convenience of most experimental scientists, a web-server for EnhancerPRED was available at http://server.malab.cn/EnhancerPRED/.

## Materials and Methods

### Datasets

In this study, we used the recently constructed dataset reported elsewhere^17^. As described previously^25^,^26^, the benchmark dataset was constructed based on information on the chromatin state of nine cell lines, namely, H1ES, K562, GM12878, HepG2, HUVEC, HSMM, NHLF, NHEK and HMEC. To be consistent with the length of nucleosome and linker DNA, fragments of 200 base pairs (bp) in length were extracted from these nine cell lines. After removing pairwise sequence identity with threshold 0.8 and randomly selecting, we obtained a dataset containing 742 strong enhancers, 742 weak enhancers (positive training dataset) and 1484 nonenhancers (negative training dataset)^17^.

### Feature extraction derived from sequences

In order to get more available information from sequences, we extracted features from overall and partial two aspects. Bi-profile Bayes was used to reflect the distribution of nucleotides in the whole sample, while the nucleotide composition and pseudo-nucleotide composition were applied to reflect the composition of nucleotides and nucleotides’ intrinsic correlation in one DNA sample. Their definitions are as following.

#### Bi-profile Bayes (BPB)

The recently proposed BPB^27^ outperforms other methods because of its consideration of information from both positive and negative training samples. It has been applied successfully to many fields of bioinformatics, such as predicting protein methylation sites^27^, caspase cleavage sites^28^, mitochondrial proteins of malaria^29^, type III secreted effectors^30^ and RNA methylation^31^.

Considering a DNA peptide sequence S consisting of A, G, C and T, we encoded this sequence into a probability vector V = (*p*_*1*_*, p*_*2*_ *,…, p*_*n*_*, p*_*n+1*_*, …, p*_*2n*_), where *p*_*i*_ (*i* = *1, 2, …, n*) denotes the posterior probability of each nucleotide at the *i-*th position in positive samples and *p*_i_ (*i* = *n* + *1, n* + *2, …. 2n*) denotes the posterior probability of each nucleotide at the *i*-th position in negative samples (n is the length of one peptide sequence and n = 200 in the present study). When the number of samples is large enough, the frequency approximates the probability. Therefore, the posterior probability of positive and negative samples was calculated as the occurrence of each nucleotide at each position in the positive and negative training datasets, respectively^27^. In this study, the number of features was 400, and the 1–200 features were derived from the overall characteristics of positive samples, while the 201–200 features were derived from the overall characteristics of negative samples.

#### Nucleotide composition (NC) and pseudo-nucleotide composition (PseNC)

The concept of pseudo-amino acid composition or Chou’s PseAAC was proposed in 2001, and has penetrated rapidly into almost all fields of computational proteomics^32^,^33^,^34^. For a brief introduction to Chou’s PseAAC and its recent development and applications, a comprehensive review is available^35^. Recently, the concept of the pseudo-component approach was further employed in the fields of computational genetics and genomics^36^,^37^,^38^,^39^,^40^,^41^,^42^,^43^,^44^,^45^.

In this study, the nucleotide composition (NC) was calculated as a feature vector. The dimension of the NC feature vector is 4, defined as follows:


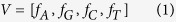


where *f*_*i*_ represents the normalized frequency of occurrence of the *i*-th nucleotide (i = A, T, G, C) in a DNA sample.

If only using NC to extract features, the sequence-order information hidden in DNA samples would be lost, markedly reducing the quality of prediction^36^,^37^,^38^,^39^,^40^,^41^,^42^,^43^,^44^,^45^. Nucleotide triplets form codons within coding regions, each of which specifies a particular amino acid. Therefore, instead of considering dinucleotide composition, the occurrence frequencies of the 3 nearest residues (trinucleotide) along the DNA sequence were adopted to stand for one DNA fragment. The corresponding feature vector thus contains 4^3^ components, as given by:


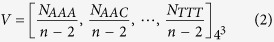


where n was the length of DNA sample and *N*_*i*_ represents the occurrence number of the *i*-th trinucleotide (i = AAA, AAC, …, TTT) in the DNA sequence. For convenience, we named 3 nearest residues (or 3-mer) composition as the pseudo-nucleotide composition (PseNC), in accordance with previous work^35^,^36^,^37^,^38^,^39^,^40^,^41^,^42^,^43^,^44^,^45^.

### SVM implementation and parameter selection

SVM is a set of related supervised learning methods used for classification and regression based on statistical learning theory. This method has been shown to be powerful in many fields of bioinformatics^29^,^30^,^31^,^32^,^46^,^47^. In this study, SVM was trained with the LIBSVM package^48^ to build the model and perform the prediction. The radial basis function kernel was used in our SVM model. For different input features, penalty parameter C and kernel parameter γ were optimized using SVMcg in the LIBSVM package based on 15-fold cross-validation. The final parameters C = 0.35355 and γ = 0.03125 were assigned for the detection of enhancers and nonenhancers, while C = 0.35355 and γ = 1.4142 were assigned for the detection of strong enhancers and weak enhancers.

### Feature selection via F-score

As heterogeneous features are often redundant and noisy, we performed feature selection to pick up the most important features by a feature selection tool known as F-score^49^,^50^. The F-score of the *i-*th feature is defined as:


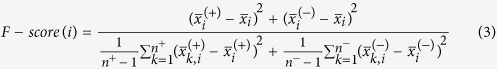


where *x*_*i*_, 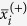 and 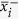 are the average values of the *i-*th feature in whole, positive and negative datasets, respectively. *n*^**+**^ denotes the number of positive data, *n*^*−*^ denotes the number of negative data, 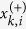 denotes the *i-*th feature of the *k-*th positive instance and 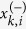 denotes the *i-*th feature of the *k-*th negative instance. A greater F-score indicates a greater difference between two classes and reflects more reliable classification. The flowchart of the features selection was supplied in Fig. S1.

## Additional Information

**How to cite this article**: Cangzhi, J. and He, W. EnhancerPred: a predictor for discovering enhancers based on the combination and selection of multiple features. *Sci. Rep.* **6**, 38741; doi: 10.1038/srep38741 (2016).

**Publisher's note:** Springer Nature remains neutral with regard to jurisdictional claims in published maps and institutional affiliations.

## Supplementary Material

Supplementary Information

## Figures and Tables

**Table 1:** The best performance of EnhancerPred in jackknife test.

Layer	Features	Sn(%)	Sp(%)	Acc(%)	MCC
I	BPB(104)	70.96	83.02	76.99	0.54
	BPB(104) + NC(1)	71.02	83.02	77.02	0.54
	BPB(104) + NC(1) + PseNC(9)	71.97	82.82	77.39	0.55
II	BPB(89)	69.41	65.23	67.32	0.35
	BPB(89) + PseNC(10)	71.16	65.23	68.19	0.36

**Table 2:** Comparison of different classifiers for identifying enhancers and their strength.

Layer	Classifier	Sn(%)	Sp (%)	Acc(%)	MCC
I	KNN(23)	59.43	89.82	74.63	0.52
	Naïve Bayes	75.27	76.42	75.84	0.52
	Random Forest	73.25	76.75	75.00	0.50
	Ensembles for Boosting	73.99	75.07	74.53	0.49
	GBDT	75.81	73.45	74.63	0.49
	libD3C	66.44	63.41	64.93	0.30
	SVM	71.97	82.82	77.39	0.55
II	KNN(45)	67.79	64.56	66.17	0.32
	Naïve Bayes	74.93	58.76	66.85	0.34
	Random Forest	66.85	59.16	63.01	0.26
	Ensembles for Boosting	69.68	61.05	65.36	0.31
	GBDT	60.51	68.19	64.35	0.29
	libD3C	55.53	54.18	54.85	0.10
	SVM	71.16	65.23	68.19	0.36

**Table 3:** Results of the comparison of EnhancerPred with the predictor iEnhancer-2L on the jackknife test.

Layer	Methods	Sn(%)	Sp(%)	Acc(%)	MCC
I	iEnhancer-2L	78.09	75.88	76.89	0.54
	Our method	71.97	82.82	77.39	0.55
II	iEnhancer-2L	62.21	61.82	61.93	0.24
	Our method	71.16	65.23	68.19	0.36
