# Supplementary material for: EnhancerPred: a predictor for discovering enhancers based on the combination and selection of multiple features
Source: Sci Rep. 2016 Dec 12;6:38741. doi: 10.1038/srep38741 (PMC5150536; doi:10.1038/srep38741)
Supplement: Supplementary Information [file srep38741-s1.pdf]

EnhancerPred: a predictor for discovering enhancers based on the combination and selection of multiple features

CangzhiJia*,Wenying He

Department of Mathematics, Dalian Maritime University, No. 1 Linghai Road, Dalian 116026, China;

*Corresponding author. Email: cangzhijia@dlmu.edu.cn

Table S1 F-score values of nucleotide composition.

Nucleotide	F-score
A	0.158
T	0.193
G	0.234
C	0.236

Table S2 104 features from BPB combined with NC.

Features	Sn(%)	Sp(%)	Acc(%)	MCC(%)
BPB(104)+f_C	71.02	83.02	77.02	0.5444
BPB(104)+f_C+ f_G	71.36	82.41	76.89	0.5410

Table S3 Predictive performances of models trained on features selected by different F-score thresholds of PseNC and BPB(104)+f_C for identifying enhancers and non-enhancers.

Threshold	Number of features from PseNC	Sn(%)	Sp(%)	Acc(%)	MCC
0.01	55	71.29	82.95	77.12	0.5462
0.02	49	71.29	82.88	77.09	0.5455
0.03	47	71.36	82.88	77.12	0.5461
0.04	39	71.36	82.75	77.06	0.5446
0.05	32	71.43	82.82	77.12	0.5460
0.06	27	71.70	82.82	77.26	0.5485
0.07	22	71.63	82.88	77.26	0.5486
0.09	20	71.70	82.88	77.29	0.5493
0.1	17	71.90	82.48	77.19	0.5469
0.12	14	71.56	82.82	77.19	0.5473
0.14	12	71.70	82.88	77.29	0.5493
0.15	11	71.77	82.82	77.29	0.5492
**0.16**	**9**	**71.97**	**82.82**	**77.39**	**0.5511**
0.17	8	71.70	82.88	77.29	0.5493
0.18	6	71.50	82.88	77.19	0.5474
0.19	4	71.36	82.88	77.12	0.5461
0.2	3	71.36	82.88	77.12	0.5461

Table S4 Predictive performances of models trained on different number of features from PseNC combined with BPB(104)+f_C for identifying enhancers and non-enhancers.

Number of features from PseNC	Sn(%)	Sp(%)	Acc(%)	MCC
22	71.63	82.88	77.26	0.5486
20	71.70	82.88	77.29	0.5493
18	72.04	82.55	77.29	0.5489
16	71.90	82.48	77.19	0.5469
14	71.56	82.82	77.19	0.5473
12	71.70	82.88	77.29	0.5493
11	71.77	82.82	77.29	0.5492
10	71.77	82.82	77.29	0.5492
**9**	**71.97**	**82.82**	**77.39**	**0.5511**
8	71.70	82.88	77.29	0.5493
6	71.50	82.88	77.19	0.5474
4	71.36	82.88	77.12	0.5461

Table S5 Predictive performances of models trained on features selected by different F-score thresholds of PseNC and BPB(89) for identifying strong enhancers and weak enhancers.

Threshold	Number of features from PseNC	Sn(%)	Sp(%)	Acc(%)	MCC
0.05	6	69.81	64.29	67.05	0.3415
0.04	7	69.81	63.88	66.85	0.3375
**0.03**	**8**	**70.35**	**65.23**	**67.79**	**0.3563**
**0.02**	**16**	**71.02**	**64.15**	**67.59**	**0.3526**
0.01	25	70.49	63.21	66.85	0.3378

Table S6 Predictive performances of models trained on different number of features from PseNC combined with BPB(89) for identifying strong enhancers and weak enhancers.

Number of features from PseNC	Sn(%)	Sp(%)	Acc(%)	MCC
9	70.75	65.36	68.06	0.3617
**10**	**71.16**	**65.23**	**68.19**	**0.3645**
11	71.02	64.69	67.86	0.3579
12	71.29	64.29	67.79	0.3567
14	71.16	63.88	67.52	0.3513

Table S7 Comparison prediction results of different k neighbors.

k	Sn(%)	Sp(%)	Acc(%)	MCC
7	59.10	86.19	72.64	0.4704
15	58.96	88.68	73.82	0.4990
**23**	**59.43**	**89.82**	**74.63**	**0.5170**
31	58.49	89.89	74.19	0.5096
25	59.10	89.29	74.19	0.5075
27	58.96	89.35	74.16	0.5071

Table S8 Comparison prediction results of different k neighbors.

k	Sn(%)	Sp(%)	Acc(%)	MCC
11	60.24	61.46	60.85	0.2170
19	63.07	62.94	63.01	0.2601
27	65.63	63.34	64.49	0.2898
35	65.63	63.75	64.69	0.2939
43	67.12	64.15	65.63	0.3128
**45**	**67.79**	**64.56**	**66.17**	**0.3236**
47	67.25	64.42	65.84	0.3168

Figures

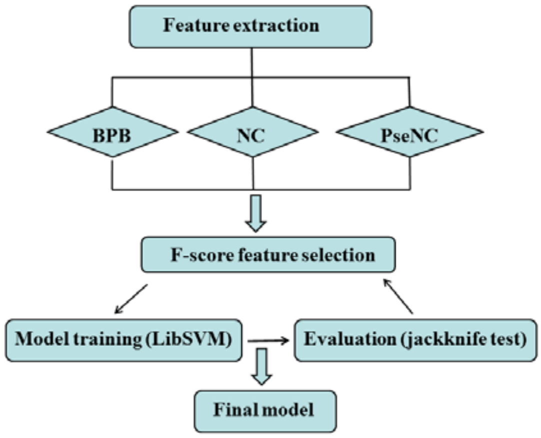

Fig. S1 The flowchart of the features selection.
